# Application of novel CAR technologies to improve treatment of autoimmune disease

**DOI:** 10.3389/fimmu.2024.1465191

**Published:** 2024-10-09

**Authors:** Abigail Cheever, Chloe C. Kang, Kim L. O’Neill, K. Scott Weber

**Affiliations:** Department of Microbiology and Molecular Biology, Brigham Young University, Provo, UT, United States

**Keywords:** CAR T cell, autoimmunity, immunotherapy, logic-gated CAR, protein-secreting CAR, modular CAR, CAR Treg, CAAR T cell

## Abstract

Chimeric antigen receptor (CAR) T cell therapy has become an important treatment for hematological cancers, and its success has spurred research into CAR T cell therapies for other diseases, including solid tumor cancers and autoimmune diseases. Notably, the development of CAR-based treatments for autoimmune diseases has shown great progress recently. Clinical trials for anti-CD19 and anti-BCMA CAR T cells in treating severe B cell-mediated autoimmune diseases, like systemic lupus erythematosus (SLE), have shown lasting remission thus far. CAR T cells targeting autoreactive T cells are beginning clinical trials for treating T cell mediated autoimmune diseases. Chimeric autoantigen receptor (CAAR) T cells specifically target and eliminate only autoreactive B cells, and they have shown promise in treating mucosal pemphigus vulgaris and MuSK myasthenia gravis. Regulatory CAR T cells have also been developed, which show potential in altering autoimmune affected areas by creating a protective barrier as well as helping decrease inflammation. These new treatments are only the beginning of potential CAR T cell applications in treating autoimmune disease. Novel CAR technologies have been developed that increase the safety, potency, specificity, and efficacy of CAR T cell therapy. Applying these novel modifications to autoimmune CARs has the potential to enhance the efficacy and applicability of CAR therapies to autoimmune disease. This review will detail several recently developed CAR technologies and discuss how their application to autoimmune disease will improve this emerging field. These include logic-gated CARs, soluble protein-secreting CARs, and modular CARs that enable CAR T cell therapies to be more specific, reach a wider span of target cells, be safer for patients, and give a more potent cytotoxic response. Applying these novel CAR technologies to the treatment of autoimmune diseases has the potential to revolutionize this growing application of CAR T cell therapies.

## 1 Introduction

### 1.1 Background on CAR T cells

Chimeric Antigen Receptor (CAR) T cell therapy is a recent immunotherapy that harnesses the immune system to specifically eliminate disease-causing cells. Thus far, they have been primarily used in treating hematological cancers, though research is ongoing to apply this treatment strategy to other diseases (1). CAR T cells are genetically engineered with a novel receptor that binds to a specific antigen and then activates the T cells innate killing mechanisms. CAR T cells currently used in clinical applications are produced using an exogenous process, where T cells are isolated from a patient, genetically engineered with a CAR using lentivirus, tested and expanded, and then transfused back into the same patient (2). Other mechanisms of CAR manufacturing and administration are currently being developed. For example, CRISPR/Cas9 is being used to introduce the CAR into the genome of T cells in a specific location to remove the potential of knocking out an essential gene due to random insertion of the CAR into the genome of the T cell during lentiviral transduction (3, 4). There is also research ongoing to enable *in vivo* CAR manufacturing, which removes the time consuming and costly step of T cell isolation and expansion. These *in vivo* CAR T cells use targeted lentivirus or nanoparticles to genetically engineer the patients T cells with the CAR without removing the T cell from the patient (5, 6).

The CAR binding region is central to the specificity and activation potential of CAR T cells. The binding region of a CAR T cell is traditionally a single chain variable fragment (scFv) comprised of the variable regions of the heavy and light chains of an antibody connected with a peptide linker (7). This enables the CAR T cell to have the specificity of an antibody while using a smaller binding domain that can be surface expressed more easily. The scFv is connected by a hinge region to the transmembrane domain. Hinge regions vary across CAR constructs, but research has shown that the different properties of hinge regions, including rigidity, amino acid pattern, and distance between the target epitope and the cell membrane significantly impact the strength of signaling and the cell’s ability to reach certain antigens (8–11). The transmembrane domain is responsible for localizing the CAR in the membrane of the T cell, and different transmembrane domains are known to affect the surface expression of the CAR (8). Connected to the transmembrane domain are the signaling domains, which enable a cytotoxic response of the CAR T cell against the cell it has bound to. The most common signaling domains are CD3ζ as the primary signaling domain, with CD28 or 4-1BB as a costimulatory domain; however, other signaling domains are also being used in CAR T cell research to alter the signaling function of the CAR T cells (12–14). CAR T cells exhibit cytotoxicity against the cell they are bound to through Fas/FasL interaction and through directional release of perforin and granzymes (15). The activation of the CAR T cells also causes other cytotoxic effects, including the release of cytokines and other typical cytotoxic responses inside of the T cell (15, 16). Unlike standard T cell receptors, which require multiple binding interactions with MHC, antigen, costimulatory domains, and cytokines to initiate a full-scale cytotoxic response, CAR T cells achieve full activation upon binding of only the CAR, because CARs are a synthetic construct which combine the different activation domains (17). However, CAR T cells follow the pattern of traditional TCR activation by forming an immunological synapse, where multiple receptors migrate together and bind to antigens on the target cell and creates enough activation to elicit a full scale cytotoxic response (18).

The concept of a CAR T cell was originally developed in 1987 as a combination of B and T cell receptors, though it wasn’t until later that they were proposed as a potential immunotherapy (19, 20). The first effective CAR T cells, second generation CARs, were created by combining the base CD3ζ CAR with a CD28 costimulatory domain in 1998 (21). These CAR T cells were then developed to eliminate leukemia cells; shown first *in vitro* and then in a mouse model (22, 23). In 2008 the first Phase I clinical trials for CD19 CAR T cell therapy began (NCT01493453, NCT00924326), leading to FDA approval in 2019 (24). CAR T-cell therapies were first FDA approved for treatment of pediatric and young adult acute lymphoblastic leukemia (ALL) and later other B cell malignancies (25). There are currently six FDA approved CAR T-cell therapies, all for hematological cancers (13, 26, 27). The overall response rate for patients treated across all FDA approved CAR T cells therapies is ~80%, quickly making CAR T therapy a valuable treatment option for patients with relapsed and refractory hematological cancers (27, 28). Currently, FDA approved CAR T cell therapies target B cell cancers using scFvs that target B cell surface receptors, including CD19 and BCMA (13). Thus, treatment with CAR T cell therapy eliminates all B cells, often completely eradicating the patient’s B cell cancer and allowing naïve B cell populations to return, though leaving the patient partially immunosuppressed (26, 29).

CAR T cells have had great success in treating hematological cancers but not without side effects. The most prevalent of these side effects is cytokine release syndrome (CRS) where the CAR T cells overstimulate the immune system, resulting in a disease that can progress to neurotoxicity, a life-threatening state (30). CRS is commonly known to be caused by overproduction of inflammatory cytokines, with IFN-γ and IL-6 specifically having been found to play a major role in severe CRS development (30–33). Though the effect of CRS on patients receiving CAR T cell therapy is evident, CRS is being combatted through various methods, the most standard of which is administration of a blocking antibody against the Il-6 receptor to manage toxicity without affecting CAR T cell efficacy (34).

Due to the great success of CAR T cells in hematological cancers, research is ongoing to develop CAR T cells that treat other diseases as well. The majority of these studies are focused on making CAR T cell therapies to treat solid tumor cancers; however, the nature of solid tumors introduces many roadblocks which still need to be overcome to make their treatment viable (35). A much smaller subset of CAR T cell research is focused on applying this immunotherapy method to non-cancerous diseases, primarily to autoimmune diseases.

### 1.2 CAR T cells being used to treat autoimmune disease

CAR T cell therapies used for treatment of autoimmune diseases are an emerging field that is showing promise in providing lasting treatments to patients. Four main strategies have been employed in using CAR T cells for autoimmune disease. First, a B cell depletion strategy using anti-CD19 and anti-BCMA CAR T cells, second, an autoreactive T cell elimination strategy, third, a targeted autoreactive B cell elimination strategy using chimeric autoantigen receptor (CAAR) T cells, and fourth, a regulatory CAR T cell strategy.

#### 1.2.1 B cell depletion strategy

**Figure 1:**
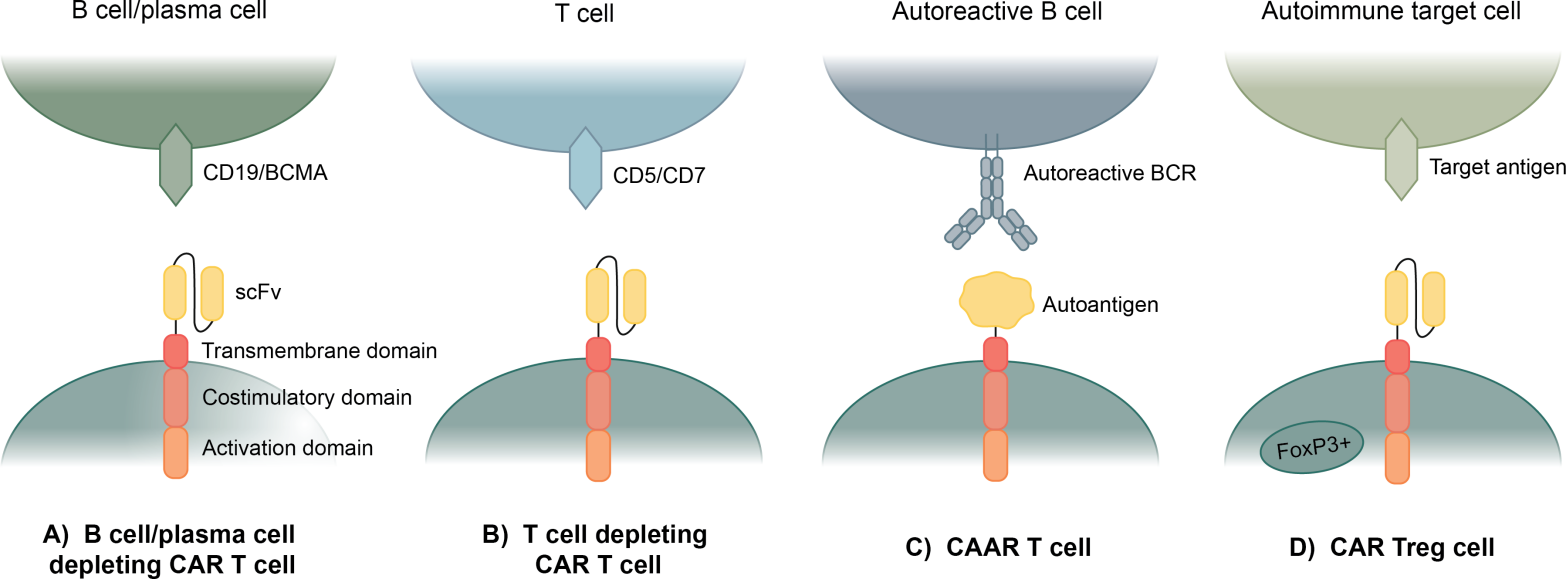
Current applications of CAR T cell therapy to autoimmune disease. **(A)** Anti-CD19 and anti-BCMA CAR T cells use an anti-CD19 or anti-BCMA scFv to eliminate all B cells or plasma cells. **(B)** Anti-CD5 and anti-CD7 CAR T cells use an anti-CD5 or anti-CD7 scFv to eliminate most T cells. **(C)** Chimeric autoantigen receptor (CAAR) T cells specifically eliminate autoreactive B cells by expressing an autoantigen as the binding domain of the CAR, which enables binding to autoreactive BCRs. **(D)** Regulatory CAR T cells (CAR Treg) bind to a target antigen in a diseased area and exhibit a regulatory T cell response to suppress the hyperactive autoimmune response.

B cell-depleting CAR T cells have been repurposed as a treatment for B cell-mediated autoimmune diseases, with the first using an anti-CD19 scFv CAR for systemic lupus erythematosus (SLE) (**Figure 1A**). Several B cell depletion drugs and monoclonal Abs have been used to eliminate B cell populations in SLE patients, but they have only been partially effective at B cell depletion (36–38). Anti-CD19 CAR T cells have been repurposed for B cell depletion in SLE patients, as shown by completely depleted B cell populations in two SLE mouse models (39). Ab levels were also depleted, and clinical manifestation of SLE in the mice was greatly improved as well (40). In a Phase I clinical trial, anti-CD19 CAR T cells were safe and well tolerated, and after a median of three months following treatment, all patients achieved full SLE remission (41). The patients also showed a return of naïve B cell populations about 100 days after treatment, allowing patients to reform B cell protective immunity, though patients have not shown a return of autoreactive B cell populations as far as currently seen (41).

A patient with severe multidrug-resistant dermatomyositis (anti-synthetase syndrome) was also treated with anti-CD19 CAR T cells and achieved similar results to the SLE patients treated prior (42). An additional case study highlighted CD19 CAR T cell administration to a patient with stiff-person syndrome (SPS) (43). Following more than six months of monitoring after a single infusion of anti-CD19 CAR T cells, the patient demonstrated deep B cell depletion and modest improvement of symptoms (43). After CAR T cell administration, the patient experienced low-grade CRS which was resolved upon treatment (43). Case studies have also been performed using anti-CD19 CAR T cells in conjunction with organ transplants to prevent potential Graft Versus Host Disease (GVHD). Not all uses have been successful, though some cases have been successful in treating GVHD following heart and kidney transplants (44–46).

Recently, results were published from a case series that administered an anti-CD19 CAR T cell therapy to 8 patients with SLE, 3 patients with idiopathic inflammatory myositis, and 4 patients with systemic sclerosis (47). The patients were all given one dose of anti-CD19 CAR T cell therapy and followed for up to two years. All patients in the study achieved remission and significant decrease in disease scores according to the clinical guidelines for each disease (47). Patients quickly demonstrated deep B cell depletion after a mean of 5.9 days. Naïve B cell populations returned in 14/15 patients after a mean of 112 days, though one patient has not yet seen any return of B cells. Patients also showed a long-term absence of autoantibodies after 1 year of treatment, suggesting that the autoreactive B cell populations do not reappear following B cell repopulation.

In many autoimmune diseases, plasma cells have been shown to play a significant role in pathogenesis (48). CAR T cell therapies have been designed to target BCMA instead of CD19 to more effectively target these plasma cell populations. Anti-BCMA CAR T cells therapies have been FDA-approved for refractory multiple myeloma, thus, increasing the number of clinical trials for anti-BCMA CAR T cell therapies being applied in other applications (49, 50). Anti-BCMA CAR T cell clinical trials are especially focused on central nervous system autoimmune disorders like Neuromyelitis Optica (NCT06279923, NCT06485232, NCT06249438, NCT04561557, NCT05828212) (51). Some successes have been reported for the safety of these treatments and a decrease in neuroinflammation following anti-BCMA CAR T cell therapy (51–53).

Many further clinical trials are underway using anti-CD19 and anti-BCMA CAR T cells for treatment of many B cell-mediated autoimmune disorders (**Figure 1A**). The following table summarizes all the current clinical trials for CAR T cell therapies in the treatment of autoimmune diseases (**Table 1**). As of August 8, 2024, all of these clinical trials are in Phase I or combined Phase I/II. In total, there are 67 ongoing clinical trials for anti-CD19 and anti-BCMA CAR T cell therapies for the treatment of autoimmune disease. These span across 26 different autoimmune and related disorders which are being tested in these clinical trials. The vast majority of these clinical trials have been initiated within the past year, showing the recent development and rapid growth of this field.

**Table 1:** List of all CAR T cell trials for autoimmune disease.

Treatment type	Disease	Clinical trial ID
Anti-CD19/BCMA CAR T cell therapy	Systemic Lupus Erythematosus (SLE)	NCT06279923, NCT06428188, NCT06056921, NCT06347718, NCT06373081, NCT06249438, NCT06420154, NCT06350110, NCT06513429, NCT05859997, NCT06417398, NCT06361745, NCT06222853, NCT06340750, NCT06465147, NCT03030976, NCT06316791, NCT06340490, NCT05474885, NCT06294236, NCT05858684, NCT05846347, NCT05988216, NCT06310811, NCT06462144, NCT06121297, NCT06530849, NCT06429800, NCT06153095, NCT06297408, NCT06333483
	Lupus Nephritis	NCT05085418, NCT06350110, NCT05938725, NCT06342960, NCT06285279, NCT06497361, NCT06497387, NCT06277427, NCT06121297, NCT06429800, NCT06153095
	Systemic sclerosis	NCT06279923, NCT06347718, NCT06373081, NCT06420154, NCT06350110, NCT05859997, NCT06417398, NCT06361745, NCT06400303, NCT06328777
	Dermatomyositis	NCT06279923, NCT06056921, NCT06347718, NCT06154252
	Polymyositis	NCT06347718
	Immune nephritis	NCT06279923
	Neuromyelitis optical	NCT06279923, NCT06485232, NCT06249438, NCT04561557, NCT05828212
	Scleroderma	NCT05085444, NCT06056921, NCT06328777
	Sjogren’s Syndrome	NCT05085431, NCT06056921, NCT06373081, NCT06420154, NCT06350110, NCT05859997, NCT06417398, NCT06361745
	Anti-Neutrophil Cytoplasmic Antibody-Associated (ANCA) Vasculitis	NCT06056921, NCT06350110, NCT06373081, NCT06420154, NCT05859997, NCT06508346, NCT06285279, NCT06294236, NCT06277427, NCT06462144
	Immune Thrombocytopenia (ITP)	NCT06352281, NCT06417398, NCT06519565
	Myasthenia Gravis	NCT06485232, NCT04561557, NCT05828225, NCT06193889, NCT06371040, NCT06359041, NCT06419166
	Multiple Sclerosis	NCT06485232, NCT06249438, NCT06451159, NCT06138132, NCT04561557, NCT06384976
	Chronic Inflammatory Demyelinating Polyradiculoneuropathy	NCT06485232
	Inflammatory Myopathy	NCT06373081, NCT06420154, NCT05859997, NCT06417398, NCT06361745, NCT04561557, NCT06462144, NCT06154252
	Antiphospholipid Syndrome	NCT06373081, NCT06420154, NCT05859997
	Immune-Mediated Necrotizing Myopathy	NCT06249438, NCT04561557, NCT06154252
	Autoimmune Hemolytic Anemia	NCT06231368, NCT05263817, NCT06212154
	Granulomatous/Microscopic Polyangiitis	NCT06350110, NCT06294236
	POEMS syndrome	NCT05263817, NCT04561557
	Amyloidosis	NCT05263817
	Vasculitis	NCT05263817
	Rheumatoid Arthritis	NCT06417398, NCT06475495
	Primary Biliary Cholangitis	NCT06417398
	Autoimmune Encephalitis	NCT04561557
	Anti-synthetase syndrome	NCT06154252
	Multiple undisclosed autoimmune diseases	NCT05459870, NCT06435897, NCT06503224
Anti-CD7 CAR T cell therapy	Crohn’s Disease	NCT05239702
	Ulcerative Colitis	NCT05239702
	Dermatomyositis	NCT05239702
	Still’s Disease	NCT05239702
CAAR T cell therapy	Pemphigus Vulgaris	NCT04422912
	MuSK Myasthenia Gravis	NCT05451212
CAR Treg therapy	Hidradenitis Suppurativa	NCT06361836
	Graft Versus Host disease	NCT05234190, NCT05993611, NCT04817774

Thus, repurposed CAR T cells show promise in treating autoimmune diseases, as shown by the above trials, particularly in patients with severe and refractory autoimmune diseases. However, CD19 targeting CAR T cells eliminate all B cells for several months and anti-BCMA CAR T cells eliminate a patient’s plasma cells reserves until they can be reformed. Though B cell and plasma populations can recover after time, some previous immunity can be lost, and there is a period when patients are significantly immunosuppressed. Thus far, none of the patients in the studies and clinical trials have contracted serious illness, as far as we are aware, but measures were undertaken to protect them from infectious disease while in their immunosuppressed state. In the face of current evidence and the alternative treatments, B cell depleting CAR T cell therapies could become a valuable option for people suffering from severe B cell-mediated autoimmune diseases.

#### 1.2.2 Autoreactive T cell elimination strategy

In many autoimmune diseases, pathogenesis is instigated by autoreactive T cells instead of, or in conjunction with, autoreactive B cells and plasma cells. Targeting T cells with CAR T cell therapies is inherently more difficult than targeting B cells because of CAR T cell fratricide. However, CAR T cell therapies have been developed for severe T cell lymphomas and leukemias to target and eliminate T cells for short period of time (54). Several T cell targets, including CD5 and CD7, have been used as the target protein for these CAR T cell therapies because CD5 and CD7 have been shown to be non-essential to biological T cells function, but nearly ubiquitously expressed on T cells (**Figure 1B**) (55–57). Anti-CD5 and anti-CD7 CAR T cells have not received FDA approval yet, but progress has been made using both targets separately and in conjunction (55, 58, 59). These CAR T cells do undergo fratricide, but between genetic engineering to delete CD5 and/or CD7 from the CAR T cells and protein blockers to prevent CD5 and/or CD7 interactions, most of this fratricide can be mitigated (58).

Applying this anti-CD5 and anti-CD7 CAR T cell treatment to autoimmune diseases with a strong T cell component could be a valuable treatment option. There is currently a clinical trial ongoing which is testing anti-CD7 CAR T cells on severe forms of Crohn’s Disease, Ulcerative Colitis, Dermatomyositis, and Still’s Disease, though no results have been posted yet (NCT05239702). Anti-CD5 CAR T cells have also been shown to be capable of invading the CNS, which could be directly applicable to autoimmune diseases with nervous system involvement (55). It is likely that FDA approval will come first for the use of anti-CD5 and anti-CD7 CAR T cell therapies for T cell leukemias and lymphomas; however, this could help pave the way for its future use in treating patients with severe T cell-mediated autoimmune diseases.

#### 1.2.3 Targeted autoreactive B cell elimination strategy

Another CAR T cell-based therapy has been developed, allowing for targeted elimination of only autoreactive B cells in B cell-mediated autoimmune diseases (**Figure 1C**). CAAR T cells are designed to specifically eliminate autoreactive B cells, rather than eliminating all B cells, by replacing the scFv as the binding domain with an autoantigen that acts as bait for the autoreactive B cells which will bind via their B cell receptors (BCRs) (60). This method is dependent on the fact that B cells produce both autoantibodies and BCRs specific for the same antigens, thus by eliminating B cells with autoreactive BCRs, autoantibody levels should be eliminated (61).

CAAR T cells were pioneered for the treatment of pemphigus vulgaris (PV), a skin autoimmune disease, where autoantibodies against Dsg3, a keratinocyte adhesion protein, cause blistering and inflammation (62, 63). A panel of CAAR T cells with epitopes of DSG3 were engineered, and they showed successful cytotoxic effects against an engineered DSG3 B cell line and patient derived DSG3 B cells (62). Consideration was taken as to whether soluble anti-Dsg3 Abs would inhibit the CAAR T cell function or initiate anergy; however, in the presence of PV serum, the soluble Abs did not have an inhibitory effect on the CAAR cytotoxicity and may even have a beneficial effect in preventing exhaustion (62). In a mouse model, the same findings were replicated, and the mice showed a reduction of blistering, notably with no off-target effects recorded on healthy B cells (62). Preliminary data from PV patient blood samples quantifying dosage pharmacology were recently published, qualifying it for Phase I clinical trials (NCT04422912), which are currently ongoing (64). This team has also recently applied the same principle to the treatment of muscle-specific tyrosine kinase myasthenia gravis (MuSK MG) for MuSK-specific B cell depletion (65). These MuSK CAAR T cells effectively reduced MuSK B cell and Ab populations without affecting healthy B cells or normal Ab levels, and it has also recently begun a Phase 1 clinical trial (NCT05451212) (65).

CAAR T cells are specific in targeting autoreactive B cells, overcoming the disadvantages of complete B cell elimination and subsequent immunosuppression and making them a strong option for future treatments of B cell-mediated autoimmune diseases (66). One difficulty in this treatment strategy is that different CAAR T cells need to be engineered for each autoantigen present in an autoimmune disease. In certain diseases this is not a problem because there are few autoantigens that mediate the disease, but in other diseases, like SLE, there are many autoantigens, which would require many different CAAR T cells. Also, some autoantigens may prove difficult to engineer as the binding domain of a CAAR because of their size, folding structure, or because they are not even proteins at all.

#### 1.2.4 Regulatory CAR T cell strategy

Regulatory T cells (Treg) are a subset of CD4+ T cells that exhibit anti-inflammatory and immunosuppressive effects and are commonly characterized by expression of phenotypic markers CD25 and FOXP3 (67). Treg-based therapies have been explored as a potential treatment for autoimmune diseases because of their ability to counteract the chronic inflammation associated with autoimmune disease (68). Some of these treatments include infusions of biologics like cytokines and drugs to support Treg function and proliferation and adoptive transfer of Tregs expanded *in vitro* (69). CAR Tregs are an application of CAR T cell therapy that target areas of chronic inflammation in autoimmune disease through an autoantigen-specific CAR construct (**Figure 1D**). The concept is quite similar to traditional CAR T cells; however, instead of inducing cytotoxic function in CD8+ T cells, the CAR activation by a specific antigen induces Treg functions to provide protection against effector T cells and other immune cells (70).

CAR Treg cells were applied in a colitis model where CAR Tregs with an scFv specific for 2,4,6-trinitrophenyl (TNP) were developed (71). These anti-TNP CAR Tregs accumulated at the gut of the mice where TNP was present and decreased inflammation (71). Another colitis application developed CAR Tregs which bound to the family of carcinoembryonic antigens (CEAs), and these cells also showed accumulation at sites which express CEAs in the gut. However, these cells were undetectable in significant quantities by day 9 post-transfusion (72). CAR Tregs also showed promise in the treatment of type-1 diabetes. Insulin-specific CAR Tregs were created, and though they were long-lived *in vivo* and delayed disease onset in NOD mice, CAR Treg therapy did not stop the onset of type-1 diabetes in mice (73). CAR Treg therapies have arguably shown the most promise as a treatment for multiple sclerosis. First, CAR Tregs targeting myelin oligodendrocyte glycoprotein (MOG) were developed, and experimental autoimmune encephalitis (EAE) mice treated with MOG CAR Tregs had significant decrease in disease scores, with most achieving recovery (74). They even rechallenged the mice with EAE-inducing inoculum, and the mice did not relapse but remained healthy (74). Another group developed CAR Tregs specific for MOG and myelin basic protein (MBP), which showed similar success in the EAE mouse model (75). One possible reason for their success is their intra-nasal delivery, which allows for lower dosage and higher accumulation rates at disease sites (74, 75).

A clinical trial is currently in progress for a CAR Treg therapy for Hidradenitis Suppurativa. Another application for CAR Treg therapies that currently has multiple clinical trials addressing it is for GVHD. In instances of transplant, an immune response against the transplanted cells or tissue can lead to rejection of the transplant (76). CAR Treg therapies can be administered in conjunction with the transplant to reduce the risk of immune-mediated rejection of the transplant. Several clinical trials are in the beginning phases of determining if this dual-treatment method is effective and safe for transplant operations (NCT05234190, NCT05993611, NCT04817774).

CAR Tregs are an innovative application of CAR T cell therapy; however, they need improvements before they can be clinically relevant. One major limitation is the safety issue of CAR Tregs losing expression of FOXP3 and the resulting anti-inflammatory phenotype when they localize in highly inflammatory regions. To counteract this, modifications are being made to induce expression of FOXP3 as part of the CAR or use CRISPR to knock out certain effector genes like IFN-γ or IL-17 (77, 78). CAR Treg persistence can also be a problem, with some reports showing that CAR Treg counts diminish rapidly *in vivo* (70, 72).

## 2 Novel CAR applications in autoimmune disease

The application of CAR T cell therapies to autoimmune disease is a promising treatment avenue, though it is still in its infancy. CAR T cell therapy is a widely researched topic, with many new developments being discovered every year. There are dozens of novel constructs that address specificity, efficacy, and safety concerns present in current CAR T cells. Some of these constructs include logic-gated CARs, which allow for increased specificity to cells by including multiple antigens; protein-secreting CARs, which can secrete soluble factors for enhanced potency; and modular CARs, which enable targeting a panel of antigens. These novel CARs have been developed with applications in cancer treatment; however, we believe their application to autoimmune disease could be just as valuable. This review will detail several of these novel CARs, their current successes in cancer treatment, and provide insight into ways they could be most valuable in treating autoimmune diseases with CAR T cell therapies.

### 2.1 Logic-gated CARs

**Figure 2:**
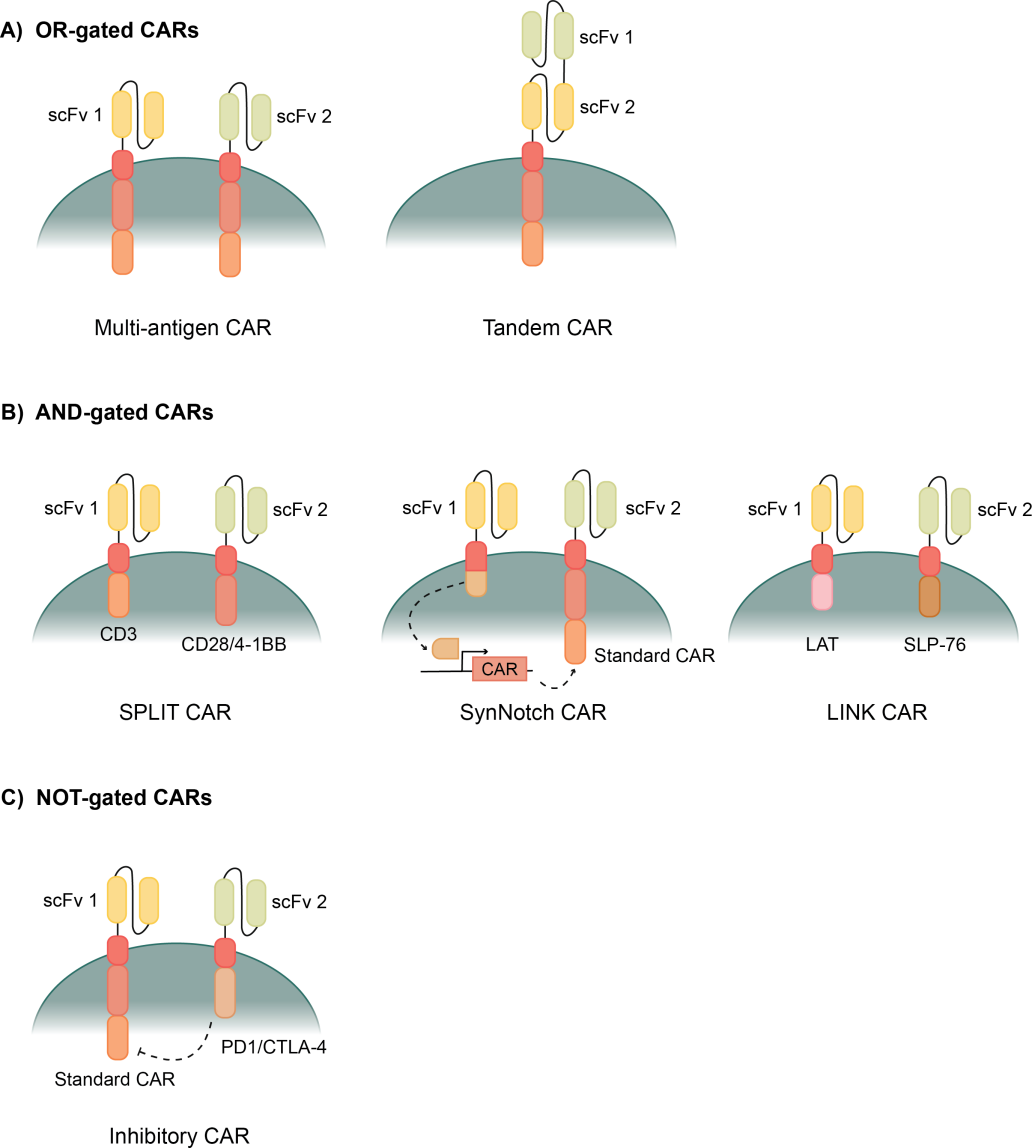
Types of logic gated CAR T cells. **(A)** OR- gated CAR T cells allow activation when exposed to one of multiple possible antigens. Multi-antigen CARs have multiple CAR constructs with different scFvs. Tandem CARs have two different scFvs attached to one CAR construct. **(B)** AND-gated CARs require binding to multiple antigens before activating the CAR. SPLIT CAR T cells have two CAR constructs that separate the primary (CD3) and costimulatory (CD28/4-1BB) activation domains. SynNotch CARs bind the first antigen, releasing a transcription factor, which then enables transcription of a second standard CAR construct. LINK CARs use downstream signaling domains, LAT and SLP-76, to give a complete AND-gated response. **(C)** NOT-gated CARs use a standard CAR and an inhibitory CAR to block a cytotoxic response when bound to certain antigens. The inhibitory CAR uses PD1/CTLA-4 to counteract the CAR’s response when its scFv is bound.

One challenge in all types of CAR T cell therapies is the selection of an appropriate target and specificity towards that target. In many cases, it can be difficult to find a surface expressed biomarker that is specific to only the diseased cells. Logic-gated CARs address this problem by applying Boolean AND, OR, and NOT logic gates to increase specificity towards a target cell (**Figure 2**). This can reduce “on-target, off-tumor” effects caused by target selection that is not completely specific to the diseased cell. It can also widen the range of targets available, as researchers can choose multiple antigens that alone are not completely specific but in combination, are entirely unique to the diseased cells.

#### 2.1.1 OR-gated CARs

OR-gated CARs are also known as multi-antigen CARs because they can recognize more than one antigen (**Figure 2A**). Recognizing more than one antigen can allow for a broader targeting of a heterogenous tumor with many biomarkers (79). OR-gated CAR T cells also help to protect against antigen escape, where the tumor cells down-regulate the target antigen, often leading to relapse, by targeting multiple markers to greatly reduce the possibility of escape (80).

One method of creating an OR-gated CAR is simply pooling multiple CARs that are specific for different single antigens (**Figure 2A**). Then the T cells express multiple CARs, specific for different antigens, and each are capable of activating the cytotoxicity of the T cells when bound (81). These CAR T cells have shown improved cytotoxicity towards tumors and are especially improved in their ability to prevent antigen escape and the associated relapse (82–84). Another method of OR-gating CAR T cells involves combining two scFvs onto the same CAR construct to create a Tandem CAR (**Figure 2A**) (85). Each ScFv binds to a different antigen, but both transmit a signal through the same transmembrane domain and activation domains. These Tandem CARs exhibit even stronger ability to prevent antigen escape because each CAR T cell is ensured to have scFvs for each antigen (86). Several studies have been run with tandem CAR T cells, showing efficient tumor clearance *in vitro* and in mouse models (85, 87). Clinical trials are also ongoing evaluating a variety of antigen combinations for various cancers (88–90). Tandem CAR T cells, in certain cases, also had stronger activation than single CAR T cells or pooled CAR T cells because antigens can bind to both scFvs on one CAR, causing a stronger signal and stronger activation response (87, 89). This is a benefit of a robust anti-tumor response; however, the response can be too strong and cause unwanted effects like exhaustion or CRS.

One limitation of OR-gated CAR T cells with multiple common antigen targets is an increased incidence of CRS. CRS is well managed in current FDA approved use of CAR T cells, but when multiple antigens can activate the CAR T cell, it is likely that patients will experience higher levels of cytokine release and more severe side effects (30). Current *in vitro* and mouse model studies suggest that this is the case; however, clinicians have developed effective ways of mitigating CRS thus far, and we hope that effective measures could be developed for making OR-gated CAR T cells safe for patients (79).

OR-gated CAR T cells have the potential to improve CAR T cell use in the treatment of autoimmune disease using multiple strategies. B cell depletion using CD19 CAR T cells has been useful in autoimmune disease, but if, in further clinical trials, CD19 antigen escape becomes a problem, an OR-gated CAR T cell with CD19 and another B cell marker like CD20 could be beneficial in delivering a complete B cell depletion (41, 82, 86, 89). There is also a possibility that CD19 B cell depletion will not eliminate the long-lived plasma cells in patients, and this could lead to a relapse of severe autoimmune disease. One method of combatting this possibility is to implement an OR-gated CAR T cell with CD19 and a marker that targets plasma cells, like BCMA and CD22 (91, 92).

Application of OR-gated CAR could also be beneficial in CAR Treg therapies for autoimmune disease. In many autoimmune diseases, there are multiple antigens under attack by the immune system. In fact, the vast majority of autoimmune diseases have at least two autoantigens. By protecting multiple antigens in the locus of an autoimmune disease using CAR Tregs, the protection and reversal of chronic inflammation has the potential to be significantly more robust. For example, CAR Treg therapies are being developed for MS that target MOG and MBP; however, an OR-gated CAR could combine those two targets for a more comprehensive therapy (75). There are also other myelin and neuronal-associated proteins that are shown to be key in driving inflammation of MS, including proteolipid protein (PLP) and myelin-associated glycoprotein (MAG), as well as several other proteins that are currently being discovered and evaluated for their consistency across patients (93, 94). Inclusion of these proteins may also improve the breadth of protection for MS patients. Rheumatoid arthritis (RA) is another severe autoimmune disease with many autoantigens, and they are often different across different forms of the disease (95). Autoantigens in this disease are often citrullinated proteins, carbamylated proteins, and the Fc region of IgG, but antibody complexes often accumulate in the synovial joints, making them the main locus of disease (96, 97). These autoantigens may not make suitable biomarkers for CAR Treg therapy, but localization of CAR Treg cells to the synovial joints could help to reverse the chronic inflammation there. Certain proteins that are exposed in damaged RA patient joints, like cartilage matrix proteins, collagen type II, and fibronectin, could be efficient targets for CAR Treg therapy (98). An OR-gated CAR Treg approach has potential to provide a broader scope of protection for the joints damaged in RA patients, and reverse the chronic inflammation there.

#### 2.1.2 AND-gated CARs

AND-gated CAR T cells have two separate constructs in the same cell with different binding domains on each construct. In theory, the T cell should only activate when both CAR constructs bind to their cognate antigens that are present on the same target cell (**Figure 2B**). Thus, AND-gated CARs are sometimes referred to as bispecific CARs because they are specific for two antigens, and binding to both antigens is required to activate the CAR T cell.

AND-gated CARs have the significant benefit of enabling specific targeting of certain cell types that do not have a single unique surface biomarker for a CAR T cell to target (99). By requiring binding to two separate antigens on one cell, more cells are targeted with greater specificity, making the CAR T cell therapy safer and more effective. Another benefit of AND-gated CAR T cells is fewer side effects caused by on-target, off-tumor effects because cells and tissues that only share one, but not both, of the target antigens are not killed.

Researchers have been attempting to develop AND-gated CARs for nearly as long as CAR T cells have existed, but creating a true AND-gate has proven difficult. First generation CAR T cells had CD3ζ as the only activation domain, and these CAR T cells could activate, but couldn’t produce a full cytotoxic response (100). It wasn’t until costimulatory domains like CD28 and 4-1BB were added to the CARs that the T cells could produce a full cytotoxic response and function as a viable therapy (14, 22). SPLIT CAR T cells separate the CD3ζ primary activation domain and the CD28 or 4-1BB costimulatory domain into two separate CAR constructs with separate scFvs (**Figure 2B**) (101). Full activation of this CAR is only achieved upon binding of both antigens; however, binding to the CD3 CAR still causes a weaker activation that can induce some cytotoxic effects (99, 101). This partial activation induced by binding to the antigen connected to CD3ζ makes SPLIT CAR T cells not truly AND-gated CAR T cells.

Synthetic Notch (SynNotch) CAR T cells are an improvement on AND-gated CAR T cells with specificity for two antigens that take a different transcription factor-mediated approach (**Figure 2B**). They are primed by binding of a first antigen to a synthetic notch CAR which releases a transcription factor that enables transcription of a traditional CAR (102, 103). Then, a second antigen can activate the CAR after it is expressed and surface localized (103). The first SynNotch CAR T cells were mediated using the transcription factor Gal4-VP64, which then drove transcription of the second CAR; however, any desired transcription factor could be used in this application (103). SynNotch CAR T cells have been applied to a variety of cancers using many target antigens (103–107). This system improves the AND-gated bispecificity, but on-target, off-tumor activation can still occur because the effect of the transcription factor can last long enough for the two binding events to occur with two different cells (108). This kinetic delay between activation by the first antigen before expression of the second CAR and its activation has been measured around 6 hours (103). Though in this time frame the two antigen interactions are often on the same cell, follow-up research has shown that this is long enough for the two antigen binding events to occur on different cells at a significant rate (109).

Recently, a novel CAR system with new signaling domains was developed which has a complete AND-gated specificity. Research began by exploring the downstream signaling molecules following a traditional T cell receptor activation, and several downstream signaling molecules were shown to function well in a CAR T cell (109). They then discovered that these TCR downstream signaling molecules could be used in combination for an AND-gated CAR, and they developed the LINK CAR system (109). LINK CAR T cells have two CAR constructs that each have a TCR signaling molecule as the activation domain, SLP-76 and LAT (**Figure 2B**) (109). When a few key mutations are made to SLP-76 and LAT, they form a true bispecific CAR where both antigens are required to bind to activate the CAR T cell (109).

Another benefit of AND-gated CARs is that they seem to preserve a healthier phenotype in the CAR T cells after prolonged activation. Syn-Notch CAR T cells retain a beneficial central memory T cell phenotype (CD45RA−CD62L+) after repeated antigen exposure, meaning that T cell differentiation in SynNotch CAR T cells was more favorable than in traditional CAR T cells (104, 106). SynNotch CAR T cells also expressed lower levels of exhaustion markers (CD39, LAG-3, PD-1, and TIM-3) after long-term antigen exposure compared to traditional CAR T cells (104, 106). Data has also shown that LINK CARs also present lower levels of exhaustion markers after consistent stimulation than traditional CAR T cells, which could be due to their use of downstream signaling molecules as activation domains (109).

The application of AND-gated CAR technology could be especially valuable in CAAR T cell treatments for autoimmune disease. CAAR T cells use an autoantigen as the binding domain of the CAR T cell to act as bait for autoreactive B cells (62). However, autoantigens often have other natural binding partners in the body, not just the autoreactive BCRs, and if the CAAR binds to other cells or soluble proteins in the body, there may be significant side effects. Another consideration is the soluble autoantibodies, which are sure to bind to the CAAR T cells. Current studies suggest that soluble autoantibodies do not have a major negative effect on the efficacy and safety of CAAR T cell therapies; however, in clinical trials, this may prove to be a limitation of the therapy (62, 65). If AND-gated CAR technologies were applied to CAAR T cell therapy for autoimmune diseases, the potential off-target effects of natural binding partners to the autoantigen and the soluble autoantibodies could be mitigated. The AND-gated CAAR T cell could have one construct with the autoantigen binding domain and another with an scFv against a B cell marker, like CD19, or a plasma cell marker like BCMA. This would ensure that the CAAR T cell only activates when bound to an autoreactive B cell. Other B cell markers could be used, and they could be tailored to specifically eliminate B cell subsets that are most influential in causing the autoimmune disease. For example, in many autoimmune diseases, long-lived plasma cells are the major producers of autoantibodies (110). Different scFvs could be used to specifically target autoreactive plasma cells, like BCMA or CD138 (48, 111).

#### 2.1.3 NOT-gated CARs

While OR and AND logic gating induces activation of the CAR T cells upon binding of a specific antigen, NOT-gated CAR T cells prevent the CAR T cell from exhibiting cytotoxic function when a specific antigen is bound (**Figure 2C**). This is a safety step that prevents CAR T cells from exhibiting on-target, off-tumor activity against cells that may express low levels of a target antigen.

Suicide switches have been engineered into CAR T cells as a safety measure to “turn off” the CAR T cells in the event of an adverse reaction, but these are irreversible and the CAR T cells are killed (112). These “off” switches are also often induced systemically, so all of the CAR T cells in a patient are turned off when it is necessary to use the suicide switch (112). Another similar safety mechanism is “off” switches for CAR T cells. These are similar to suicide switches, but their inhibitory effect does not kill the CAR T cells and is reversible (113, 114). There are several iterations of this concept, but in principle, they all follow the same pattern of a soluble factor or drug that can be given to the patient to temporarily stop the activity of the CAR T cells in the case of an adverse event (113–116). These “off switch” molecules can also be administered in varying doses, allowing for a tunable CAR T cell response (116). NOT-gated CAR T cells seek to use the principle of suicide genes and “off switches” as a safety mechanism for CAR T cells that bind to healthy cells and are causing off-tumor effects.

Inhibitory CARs (iCAR) are NOT-gated CAR T cells that were developed as a way to prevent the off-tumor effects of CAR T cell therapy. iCARs have an scFv that binds to healthy biomarkers on non-cancerous cells and that scFv connects with an inhibitory activation domain, PD-1 or CTLA-4 (**Figure 2C**) (117). Thus, when the healthy marker binds to the iCAR, the cytotoxicity of that CAR T cell is selectively inhibited (117). Studies show that the PD-1 and CTLA-4 activation through the iCAR limits the cytokine production, cytotoxicity, and proliferation of the CAR T cells when exposed to both the stimulating antigen and the inhibitory antigen (117, 118). The effect of iCAR activation is also temporary, enabling CAR T cells to be functional again after dissociating from a healthy cell, and finding its way to a cancerous cell that does not express the protected iCAR antigen (117). iCAR technology widens the potential target antigens that could be used for CAR T cell therapies, because cross-reactivity with healthy tissues can be minimized if there is another marker that makes the healthy tissues distinct from the cancer cells (117, 118).

NOT-gated CAR T cells could make the application of CAR T cell therapy to autoimmune disease safer, especially in circumstances where a desired target antigen is also present at some level on healthy cells. One limitation of NOT-gating CAR T cells is that a suitable and unique healthy antigen must be found. In many cases, this can be difficult to find with our current knowledge of surface-expressed proteins in healthy and diseased cells. There is an ongoing project to document the “surfaceome” of all unique cells and their diseased counterparts through wet-lab analysis of cells as well as computer-aided models (119–123). This knowledge will likely uncover new targets on diseased cells that could be targeted in CAR T cell therapies for cancer as well as autoimmune disease, as well as find healthy proteins that are downregulated on diseased cells that could serve in a NOT-gated CAR to improve the safety and specificity of these treatments.

### 2.2 Soluble protein secreting CARs

**Figure 3:**
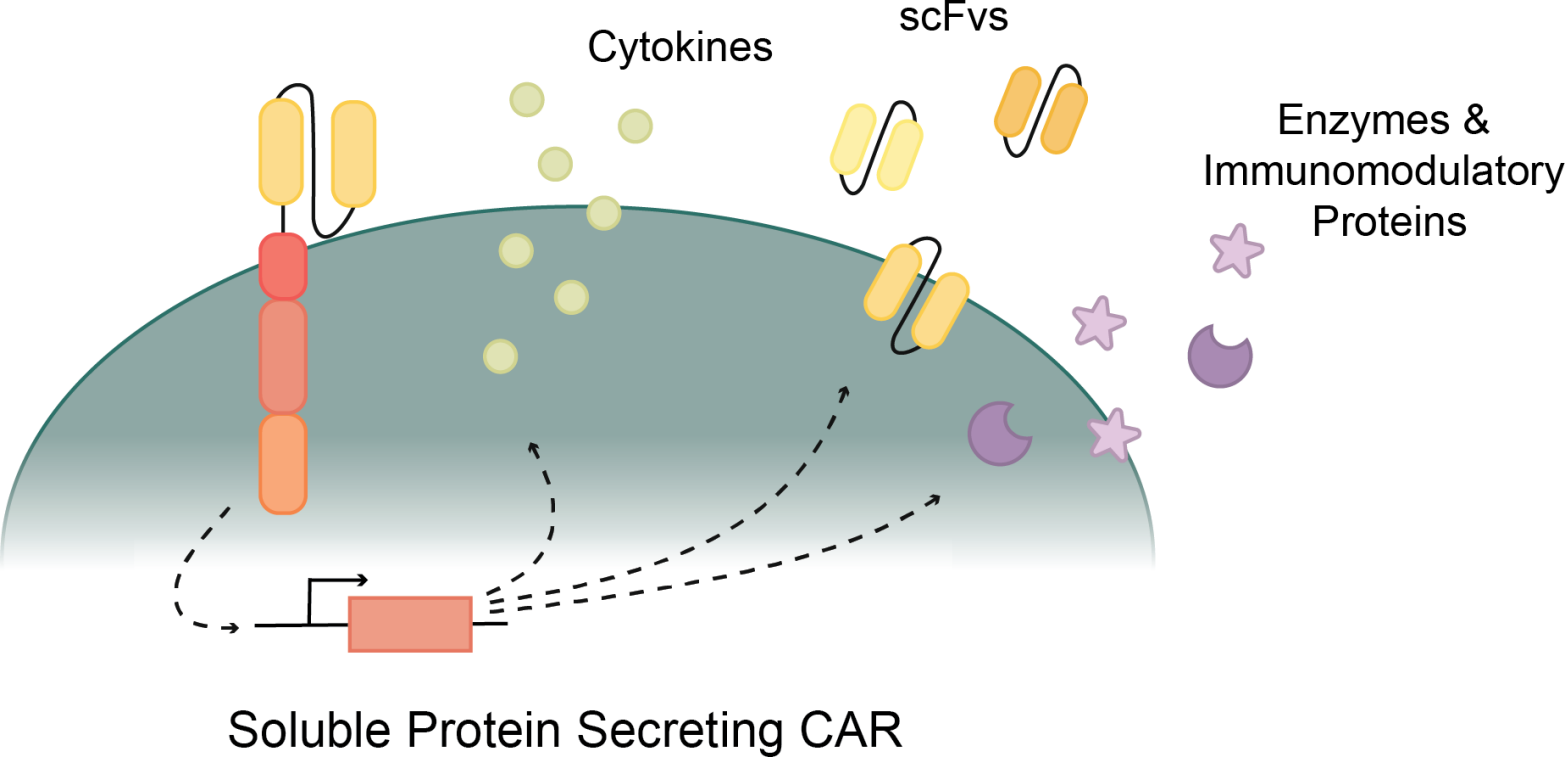
Various proteins secreted by CAR T cells to improve cytotoxicity. Cytokine secreting CARs support T cell activation and function. ScFv secreting CARs inhibit immune checkpoints to revive CAR T cells. Enzyme and Immunomodulatory protein secreting CARs weaken the tumor or disease effects and provide a more direct path for CAR T cell cytotoxicity.

Another solution to increase the efficacy of CAR T cells comes from fourth-generation or soluble protein-releasing CARs (**Figure 3**). Various cytokines, enzymes, and other soluble proteins have been known to increase the cytotoxic ability of CAR T cells (124). By engineering CAR T cells to release one of these soluble proteins upon their activation, the efficacy and viability of CAR T cells are increased. This is done in four main ways: cytokine secretion, monoclonal antibody or scFv secretion, enzyme secretion, and immunomodulatory protein secretion (124). When secreted from the CAR T cell, these soluble proteins work in a variety of ways to increase the metabolism, potency, and persistence of CAR T cells, minimize immunosuppressive effects of the tumor microenvironment (TME), and/or inhibit cytokines associated with cytokine release syndrome (CRS) (124).

#### 2.2.1 Cytokine secreting CARs

Cytokines play a key role in T cell activation and effector functions (125). T cells rely on cytokines such as IL-2, IL-7, and IL-15 to activate their cytotoxic abilities (126, 127). As signaling proteins, cytokines control inflammation in the body by activating and instructing immune cells to regulate the immune system and fight pathogens (127). Recently, researchers have been studying the benefits of combining traditional CAR T cell concepts with cytokine stimulation using various methods (125, 128). The first of these methods included CAR T cell therapy supplemented with cytokine administration (129), a cytokine or cytokine receptor expressed on the surface of the CAR T cell (130, 131), and a cytokine or cytokine receptor engineered into the CAR construct (132, 133). The combined strength of CAR T cells supplemented by the nourishment of cytokines has the potential to overcome many of the current difficulties in CAR T cell therapies. However, too many cytokines or the wrong type of cytokines can become more hurtful than helpful. Cytokines like TGF-β have been found to inhibit the activity and growth of T cells, and an overload of cytokines is known to cause CRS, both scenarios resulting in unhealthy and unproductive T cells (33, 134). Thus, in engineering cytokine-assisted CAR T cell models, it is crucial to consider the potential effects on not only the CAR T cells but the patient as well.

Earlier generations of CAR T cells relied solely on the activation from the primary and costimulatory signals, but recent studies have found that implementing a third signal increases the CAR T cell efficacy (135). These fourth-generation CAR T cells are activated by three unique signals: a CD3ζ primary signal, a CD28 or 4-1BB costimulatory signal, and a cytokine signal (128, 135). This new generation of CAR T cells harnesses the pro-inflammatory effects of cytokines by engineering the CAR T cell to secrete cytokines upon their activation (**Figure 3**). This model was inspired by studies showing that cytokines like IL-7 and IL-2 increase T cell proliferation and durability (136, 137). Cytokine secreting CAR T cells overcome limitations in survival and proliferation by providing the stimulation and activation needed to increase T cell function and health.

Many studies have had positive results in developing cytokine secreting CAR T cells with various cytokine types to enhance CAR T cell cancer therapies. In these T cells, the vector that codes for any chosen cytokine is combined with the CAR vector and then lentivirally transduced into T cells (138). These cytokine secreting CARs are often referred to as “T cells redirected for universal cytokine-mediated killing” (TRUCKs) or armored CARs, because after the CAR T cells are activated, they become self-sustaining, producing the necessary cytokines to enable themselves to continue functioning (139–145). Cytokine production will only occur once the CAR binds to its target and is activated, initiating cytokine transcription and localizing cytokine activity to limit the negative effects that more easily accompany a systemic administration of cytokines (138, 146). Following the trends of cytokines known to help T cells, cytokines that have been the most successful in supporting CAR T cell function include IL-12 and IL-18 (138, 139).

IL-12 was the first cytokine to be secreted by a CAR T cell when the concept of cytokine secreting CARs was first developed in 2011 (138, 140). IL-12 plays a key role in T cell response and regulation by improving cytotoxic T cell activation, improving Th1 helper T cell response, and inducing other inflammatory cytokines like IFN-γ and TNF-α, making it a natural choice for enhancing CAR T cells (147, 148). IL-12 has long been proven effective in increasing the efficacy of T cells through both supplementary administration and T cell surface expression of IL-12R (133, 149–152). Many more researchers have engineered IL-12 secreting CAR T cells with various target cells, resulting in consistently positive outcomes (138–140, 153). In addition to increasing the overall T cell cytotoxic response, IL-12 secretion has been shown to induce the innate immune response to target cells that have stopped presenting antigens (138) and rescue cytotoxic T cell exhaustion (152), countering two of the most consistent concerns in CAR T cell therapy applications.

IL-18 is a proinflammatory cytokine that used to be known as IFN-γ inducing factor. It activates Th1 cells to produce IFN-γ, induces innate allergic inflammation with IL-3, and increases IFN- γ and TNF- α secretion (154). IL-18 also has a significant synergistic relationship with IL-12 (155). Due to its large role in the induction of innate immune responses and its similarity and correspondence with IL-12, IL-18 is promising as a cytokine to enhance CAR T cell function. IL-18 secreting CAR T cells have proven effective in targeting myeloma cells and small cell lung cancer in mice models (156, 157). IL-18 cytokine secretion has been shown to increase the persistence of CAR T cells and enhance the anti-tumor effects of the CAR T cells (139, 156–158).

A few of the other cytokines being used in cytokine-secreting CARs include IL-2, IL-7, and IL-15 (103, 131, 159). SynNotch receptors produce various and distinct attachments to a CAR T cell, one of which is an “a la carte” cytokine profile (103). SynNotch CAR T cells secrete cytokines in response upon CD19 antigen sensing, but secretion is independent of T cell activation (103). CAR T cells engineered to express IL-7 showed superior anti-tumor capabilities to normal CARs and increased the survival rate in mouse models (159). Alternatively, IL-15Rα expressed on the surface of cells has been proven to sustain cytokine memory and contribute to T cell survival (131). While the use of each of these cytokines shows various benefits, the secretion of these cytokines will affect the patients in ways that are difficult to test in preclinical models, specifically in the likelihood of developing CRS due to an over-secretion of cytokines such as IL-6 (30). A potential solution for this is integrating an anti-inflammatory cytokine or a soluble protein that specifically inhibits the pro-inflammatory cytokines that cause CRS in CAR T cell therapy. Targeting the cytokines that lead to CRS could counter the overproduction of cytokines, increase T cell efficiency, and inhibit overstimulation and adverse effects (33). Once further trials on cytokine secreting CARs proceed, it could be beneficial to update the CD19 CAR T cell treatment to secrete IL-7 or IL-15 to support CAR T cell health and more fully deplete autoreactive B cells in autoimmune disease (66, 103, 131, 159).

Another potential application of cytokine secreting CAR T cells in autoimmunity could be engineering a CAR Treg cell that secretes an anti-inflammatory protein to counter an overreactive autoimmune response. A cytokine-secreting CAR Treg cell could resolve some the off-target effects that come with a systemic administration of cytokines as cytokines are only secreted locally. A key qualifying factor of a potential secretory protein is that it counters the autoinflammatory response both in the disease and in CRS (33). There are countless cytokines that are intricately involved in autoimmunity and inflammation, but the two that we found most promising for this purpose are IL-6 and IL-10.

IL-6 is an inflammatory cytokine that plays a major role in Rheumatoid arthritis (RA) pathogenesis and is commonly treated as a leading cause of CRS (30, 160–162). Macrophage activation by antigen-stimulated CAR T cells often results in an overproduction of IL-6, a cytokine long associated with CRS (125). IL-6 is also a key player in RA and is often countered by the anti-IL-6 drug Tocilizumab (161). Recently, a scFv of Tocilizumab has been found to produce an anti-CRS response when secreted from CAR T cells (161). Applying an IL-6 inhibitory scFv to a cytokine-secreting CAR Treg model could increase the anti-inflammatory function of the Treg while countering the risk of CRS. The effects of blocking IL-6 would be directed to the disease sites where the CAR Treg is activated, ideally mitigating the side-effects of systemic IL-6 blocking. Additionally, an anti-IL-6 CAR Treg could be an effective way of treating RA or other IL-6 mediated autoimmune diseases.

IL-10 is another inflammatory cytokine that could prove to be an opportune application of protein secreting CAR Tregs (160). IL-10 is highly elevated in CRS and in many autoimmune diseases (163–165). It is key in autoantibody production in systemic lupus erythematosus (SLE), acceleration of autoimmune diabetes, and the immunoregulatory response in RA (163–165). Applying anti-IL-10 antibody or scFv secretion from a CAR Treg would likely have anti-inflammatory effects similar to an anti-IL-6 secreting Treg, but with the potential for further implementation in a wider variety of autoimmune diseases. This anti-IL-10 secreting CAR Treg would deliver IL-10 directly to the site of autoreactivity and decrease its off-target effect. Additionally, the anti-inflammatory effects of IL-10 in RA could be harnessed to engineer an IL-10 secreting Treg that could stop destructive chronic inflammation and prevent cartilage degradation (166).

#### 2.2.2 scFv secreting CARs

Another key player in regulating T cell activation and function are checkpoint inhibitors. Immune checkpoints play a key part in immune regulation and preventing autoimmunity (167). However, cancers can upregulate checkpoint proteins to weaken immune responses and evade the immune system. Two well-known immune checkpoints in cancer are cytotoxic T-lymphocyte-associated protein 4 (CTLA-4) and programmed cell death protein 1 (PD-1). CTLA-4 is a surface-expressed protein that limits T cell activity and protects normal tissue from T cell cytotoxicity by competing with CD28 for binding with target cells (168, 169). *In vivo* research has shown that blocking CTLA-4 enhances T cell activity and can increase anti-tumor response but also presents a risk of destructive autoimmune effects due to intense lymphoproliferation (170–172). PD-1 is expressed on the surface of T cells and, upon interaction with the transmembrane ligand (PD-L1) on another cell, the T cell’s proliferation and cytotoxicity is diminished (173). By blocking the PD-1/PD-L1 pathway, immune response depletion and evasion can be decreased (174–178). Starting out as a supplement to cancer therapies, PD-1/PD-L1 blocker therapy developed into a surface-expressed protein or protein receptor and now into a protein-secreting CAR T cell (174–177). Blocking either side of the pathway is effective in blocking immune inhibition (179).

Mitigating the effect of immune checkpoints has shown great promise as a cancer immunotherapy (167, 174, 176, 177, 180–182). CAR T cells that secrete checkpoint inhibitors block the checkpoint proteins on cancer cells and immune cells, exhausted T cells are reactivated, and the durability of the T cell immune response is increased (**Figure 3**) (167). Checkpoint blockade therapies have shown their strength against cancer, but not without weakness (167, 183). While CAR T cell therapy supplemented with checkpoint inhibitors enhanced anti-tumor cytotoxicity, this method was improved upon with checkpoint inhibitor secreting CAR T cells that localize inhibition and decrease the effects and toxicities of systemic protein inhibition (174, 176, 179).

Though antibodies like anti-PD-1 and anti-PD-L1 are significant because of their broad application in various cancer types, anti-checkpoint inhibitor treatment has not always proven effective. This is mainly due to the treatment’s dependence on depleted T cell populations reactivating (167). However, the joint power of CAR T cell therapy and checkpoint inhibitor therapy is being realized in checkpoint inhibitor secreting CAR T cells (175–179, 184–186). Using CAR T cells as transport and a convenient target for checkpoint inhibitors would solve the issue of a lack of T cells and allow any depleted T cells to be reactivated by checkpoint inhibitor function. It would also increase the localization of the checkpoint inhibitor to the tumor so there are less off-target effects to the rest of the immune system across the body (117). Researchers discovered that the addition of an anti-PD-1 scFv to a CAR T cell targeting human immunodeficiency virus (HIV) increased the specific cytotoxic abilities of the CAR T cells and overall enhanced the immune response against the target HIV cells (187).

Another recent addition to protein secreting CAR T cells is anti-CD47 scFv secretion. CD47 is a surface-expressed protein that protects cells from phagocytosis by macrophages and has proven promising as an immunotherapeutic target (188). By blocking CD47 with an scFv, the protection from phagocytosis is diminished and macrophages can then come in and eliminate CD47-negative cells (189). In both *in vitro* and *in vivo* research, CD47 blocking scFv secreting CAR T cells proved effective in improving immunotherapeutic capabilities of CAR T cell treatments (188).

In the context of autoimmune therapies, a potential approach could be a CAR Treg or CAAR therapy where a blocking scFv or Ab is secreted by the activated CAR which has a blocking effect on the cytokine that is key to the pathogenesis of a specific autoimmune disease. An example would be in blocking IL-17, a pro-inflammatory cytokine that contributes to the pathogenesis of rheumatoid arthritis (190). IL-17 receptor signaling is a critical pathway in the shift from acute to chronic RA pathogenesis, and RA pathogenesis could be inhibited by an anti-IL-17 receptor secreting CAR Treg or CAAR T cell (191).

#### 2.2.3 Enzyme & immunomodulatory protein secreting CARs

Other protein-secreting CAR constructs include enzyme secreting CARs and immunomodulatory protein secreting CARs (**Figure 3**) (192, 193). Heparanase is an enzyme that degrades the extracellular matrix. This enzyme is downregulated in culture-grown T cells. By engineering heparanase into CAR T cells, extracellular matrix degradation increased, and both tumor T cell infiltration and antitumor activity improved. Another soluble protein being secreted in CAR T cell therapies is a bacterial virulence factor. CAR secretion of pro-inflammatory neutrophil-activating protein (NAP) recruited surrounding T cell responses against solid tumors (193). The combined strength of NAP and CAR resulted in slower tumor growth and higher survival rates than typical CAR T cells from the same line.

One valuable application of protein-secreting CARs to autoimmune disease could be in CARs that secrete enzymes that degrade fibrotic buildups common in several autoimmune diseases. Fibrotic autoimmune diseases are common and include rheumatoid arthritis (RA), systemic lupus erythematosus (SLE), and idiopathic pulmonary fibrosis (IPF) (194). The fibrosis in these autoimmune diseases is often a key part of the disease’s pathogenesis and leads to many of the symptoms associated with these diseases (194). Pirfenidone is an anti-fibrotic drug that has been shown effective in targeting TGF- β differentiation (195, 196). If a CAR T cell were developed that could selectively release an anti-fibrotic drug, like Pirfenidone, in the diseased locations when the CAR T cell is activated, the CAR T cell could have a dual effect where not only are autoreactive cells killed, but fibrosis could be mitigated as well.

### 2.3 Modular CAR T cells

**Figure 4:**
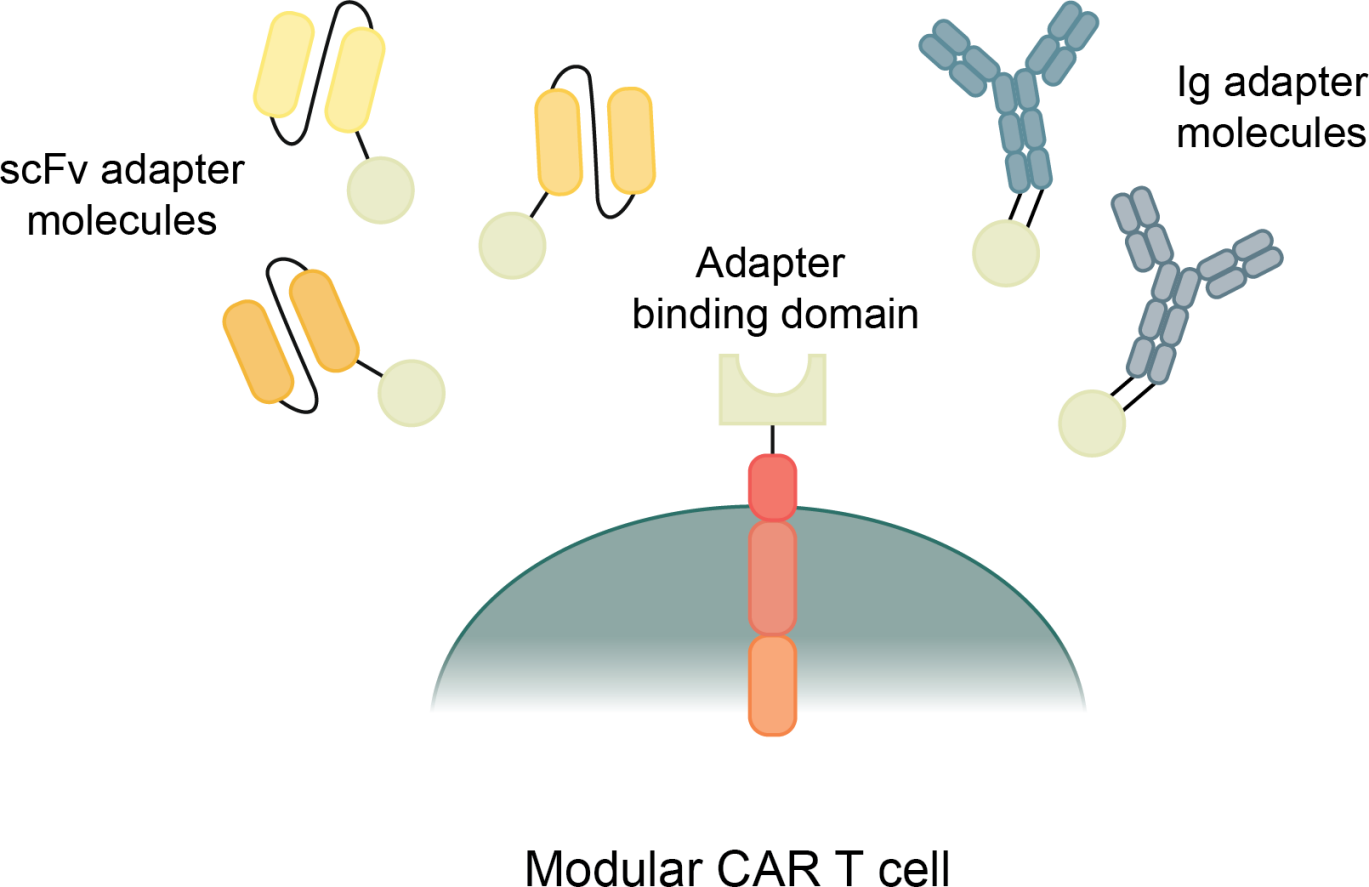
Types and mechanisms of modular CAR T cells. Modular CAR T cells have a CAR construct with a binding domain that connects to an adapter molecule. The adapter molecule binds to the target antigens and, when connecting both the antigen and the CAR, transmits a signal to initiate CAR T cell cytotoxicity.

Traditional CAR T cells are designed with a one-piece receptor that is genetically engineered into the T cell; however, new modular CARs separate the binding region from the signaling module. The Modular CAR signaling module has an extracellular portion that still serves as a binding domain, but instead of binding directly to a cancer antigen, it binds to an adapter that has the capability to redirect the CAR T cell towards specific antigens (**Figure 4**) (197). Modular CAR technology allows for more flexibility in CAR T cell dosage, tunability, multi-antigen targeting, and improved safety (197, 198).

There are many different mechanisms that are currently being used by a variety of research groups to create modular CAR T cells (197). They all share a general structure and function, though they each use different designs of adaptor molecules and different attachment mechanisms between the CAR T cell and the adapter molecule. Most modular CARs use scFv based adapter molecules to target the cancer antigens, though some use full antibody-like molecules (**Figure 4**) (197). There is even more variety in the mechanisms used for connecting the CAR signaling module to the adapter molecule. These mechanisms include avidin/biotin interaction, scFvs against tag sequences, leucine zipper molecules, covalent bonds, and enzymatic reactions (198). These many types of Modular CARs have been thoroughly described in other review articles, and for the purposes of this article, we will summarize the various modular CAR mechanisms in the following table (**Table 2**) (197, 198).

**Table 2:** Summary of modular CAR mechanisms.

Name	Adaptor molecule	Mechanism of attachment	References
Biotin binding immune receptor CAR	Biotinylated scFv	Avidin/streptavidin binding domain interacts with the biotinylation	Urbanska et al. (202) Lohmueller et al. (205)
anti-FITC CAR	FITC-tagged scFv	anti-FITC scFv CAR binds to the FITC-tagged scFv	Tamada et al. (199) Cao et al. (206) Zhang et al. (203, 207) Lee et al. (200) Ma et al. (208) Chu et al. (209) Lu et al. (210) Kim et al. (211)
SpyTag/Spy Catcher CAR	Ig with SpyTag	CAR with SpyCatcher binding domain forms covalent bond with SpyTag	Minutolo et al. (201) Liu et al. (212) (213)
Split, universal, and programmable (SUPRA) CAR	scFv with leucine zipper (zipFv)	Leucine zipper extracellular domain (zipCAR) binds to cognate leucine zipper on zipFv	Cho et al. (214, 215)
convertibleCAR	Orthogonal ligand U2S3 (MIC) fused to Ig (MICAbody)	An inert form NKG2D extracellular domain as the CAR binding domain interacts with the U2S3 ligand on the MICAbody	Landgraf et al. (216) Herzig et al. (217)
SNAP CAR	Ig with a benzylguanine (BG) motif	SNAPtag on the CAR binding domain enzymatically reacts with BG motif on Ig and enables covalent assembly	Ruffo et al. (218)
UniCAR (Anti-5B9 Tag)	5B9 tagged scFv or nanobody	Anti-5B9 scFv binds to the 5B9 tagged scFvs	Cartellieri et al. (219) Fasslrinner et al. (220) Albert et al. (221, 222) Loureiro et al. (223, 224) Mitwasi et al. (225) Feldmann et al. (226) Arndt et al. (227) Bachmann et al. (228) Stock et al. (229)
Switchable CAR	scFv tagged with a peptide neo-epitope (derived from a yeast transcription factor	scFv against the peptide neo-epitope binds to the peptide neo-epitope tagged scFvs	Rodgers et al. (230) Viaud et al. (231) Cao et al. (206)
Co-LOCKR CAR	Key protein and latch peptide with Bim tag	The key protein releases the latch peptide when bound to antigen, and then the latch peptide Bim tag bind to the Bcl-2 extracellular domain of the CAR	Lajoie et al. (232) Ng et al. (233)

Arguably, the greatest benefit of modular CAR T cells is the flexibility of multi-antigen targeting with the CAR T cells. Many adapter molecules can be produced with specificity towards a variety of antigens, but that all can activate the same CAR T cell (199). This makes it possible to target several antigens on one cancer to facilitate a more robust response and prevent antigen escape, but it also has the potential to allow for elimination of multiple antigenically different cancers with the same CAR T cells (200). Another benefit of modular CAR T cells is the tunable nature of the system. Control can be exerted by controlling the infusion dosages and timing of the adapter molecules (201). The adapter molecules have a much shorter half-life than a CAR T cell, around 90 min, which allows for more control over the duration of the cytotoxic response (200). Some CAR T cells can form memory and remain in patients forever, which can be both beneficial and harmful in certain circumstances. With a modular CAR system, the CAR T cell can form memory but will only be active when an infusion of the adapter molecule is given, providing for more control over the cytotoxic response over time (202).

Modular CAR T cells have immense potential in the treatment of autoimmune diseases, particularly because of the autoantigen diversity of many autoimmune diseases. anti-FITC modular CAR T cells have been used to treat an *in vitro* model of RA (203). Citrullinated autoantigens (citrullinated vimentin, citrullinated type II collagen, citrullinated fibrinogen and tenascin-C, and cyclocitrulline peptide-1) were produced with a FITC tag, and then anti-FITC CAR T cells were introduced (203). The FITC-tagged autoantigens directed the anti-FITC CAR T cells to the autoreactive B cells, which bound with the citrullinated autoantigens via their BCRs (203). They showed that their CAR T cells could kill the autoreactive B cell hybridomas in the presence of the autoantigen adapter molecule in a dose-dependent manner (203). This study shows that the application of modular CAR technology to CAAR T cell therapies is effective and can be very beneficial in diseases where multiple autoantigens are present. This method would also be very beneficial applied to other autoantibody-mediated autoimmune diseases like lupus, psoriasis, inflammatory bowel disease, Type-1 diabetes, and more (204). For other autoimmune diseases that are T cell and macrophage mediated, application of modular CAR technology could benefit CAR Treg therapies for these types of autoimmune diseases as well. For example, multiple scFv adapters could be produced against a variety of autoantigens in MS, and then any of the previously described CAR attachment methods could be used to create an effective multi-antigen CAR system for MS. And this method could be applied to other T cell and macrophage mediated autoimmune diseases that have multiple key autoantigens.

## 3 Discussion

Novel CAR technologies have been developed recently to improve CAR T cell function in cancers, and they are making strides toward accomplishing that goal. CAR T cells also have an important application in the treatment of autoimmune diseases, which is currently in its infancy. These autoimmune CAR T cell therapies have shown promise so far at treating severe autoimmune diseases, and the application of novel CAR developments to the treatment of autoimmune diseases has great potential in advancing this emerging field.

Logic-gated CARs allow therapies to be targeted towards a wider scope of antigens with multi-antigen OR-gated CARs, and opens the door to other antigen combinations that can more specifically target the diseased cells using AND and NOT gating. These logic-gated CARs also increase the safety of treatments by enabling more direct targeting of diseased cells. Soluble-protein secreting CARs have been developed which can significantly improve T cell proliferation and viability, which is valuable when applied to all forms of CAR T cell therapies. Other soluble protein secreting CARs can also regulate their environment by secreting scFvs which can block specific proteins, and by secreting other molecules which can degrade certain tissues. In autoimmune disease, the environment is important to the pathogenesis of the disease, making soluble-protein secreting CARs a valuable possibility. Modular CARs could also greatly impact autoimmune CAR T cell therapies because they allow for tunability of the cytotoxic response over time and more flexibility with targeting a variety of antigens at once.

We anticipate that CAR T cell therapies for autoimmune diseases will continue to be developed and become an important treatment method for patients suffering with many types of severe autoimmune diseases. Applying novel CAR technologies to autoimmune disease CAR T cell therapies will make these treatments safer, more feasible, and more effective at providing patients with long-term remission.
